# GNG12 overexpression inhibits triple-negative breast cancer cell proliferation, migration, and invasion

**DOI:** 10.1038/s41598-026-66202-z

**Published:** 2026-08-11

**Authors:** Biao-Feng Shan, Le Zhao, Tao Hu, Jing-Hao Xu, Tong-Xu Zeng, Jian-Ming Tang, Bo Yu

**Affiliations:** 1 The First Clinical Medical College of Lanzhou University, Lanzhou University, Lanzhou, China; 2 General Surgery, The Second People’s Hospital of Lanzhou, Lanzhou, China; 3 The First Clinical Medical College of Gansu University of Chinese Medicine, Lanzhou, China; 4 The Second Affiliated Hospital of Xi’an Jiaotong University (Xibei Hospital), Xi’an, China; 5 Zigong Fourth People’s Hospital, Zigong, China; 6 Breast Surgery, Zouping People’s Hospital, Binzhou, China; 7 The First Hospital of Lanzhou University, Lanzhou, China

**Keywords:** GNG12, Pan-cancer, Prognosis, Triple-negative breast cancer, Biomarkers, Cancer, Computational biology and bioinformatics, Immunology, Oncology

## Abstract

Guanine nucleotide-binding protein subunit gamma-12 (GNG12), a G protein γ-subunit, has been identified as a potential regulator of tumor biology. However, its functional relevance in triple-negative breast cancer (TNBC) remains unclear. In this study, we integrated pan-cancer transcriptomic analyses with experimental validation in TNBC models to investigate the expression patterns, clinicopathological significance, and biological roles of GNG12. GNG12 was found to be broadly downregulated across diverse cancer types, and its reduced expression was significantly associated with unfavorable patient survival outcomes. Low GNG12 expression correlated with adverse clinicopathological features in breast cancer (BRCA). Moreover, it was associated with poorer outcomes in TNBC patient cohorts. Functional assays demonstrated that GNG12 overexpression inhibited TNBC cell proliferation, migration, and invasion, whereas GNG12 knockdown exerted the opposite effects. Pathway analysis and western blotting indicated that GNG12 expression was associated with altered PI3K/AKT pathway activity. Additionally, mutations at S2 and S33—both located within predicted phosphorylation sites—were identified as candidate regulatory alterations whose functional relevance requires further validation. Moreover, GNG12 expression was positively correlated with RNA methylation-related markers and inversely associated with promoter methylation. Taken together, GNG12 was downregulated in TNBC and associated with suppression of malignant cellular phenotypes; however, its clinical significance and mechanistic relationship with PI3K/AKT signaling require further validation in larger cohorts and additional experimental models.

**Supplementary Information:**

The online version contains supplementary material available at 10.1038/s41598-026-66202-z.

## Introduction

Cancer remains the leading cause of global mortality and represents a major public health challenge^1^. Epidemiological projections estimate that the global cancer incidence will nearly double by 2070 compared with 2020 levels^2^. Among female malignancies, breast cancer (BRCA) has the highest incidence and mortality rates^3,4^. Compared with other BRCA subtypes, TNBC, in particular, is characterized by marked aggressiveness, pronounced biological heterogeneity, and poor prognosis^5^. Although targeted therapies and immunotherapies have achieved considerable breakthroughs in cancer treatment^6,7^, obstacles such as cytotoxicity and drug resistance remain major barriers to drug development and clinical application^8,9^. For most TNBC patients, chemotherapy remains the primary treatment modality across all disease stages^10^. These limitations underscore the need for a deeper understanding of the molecular alterations associated with TNBC progression, as well as for the identification of candidate molecules that may inform future biological and translational studies.

GNG12, recently recognized as a tumor-associated molecule, has attracted increasing scientific interest^11,12^. Although the functional mechanisms of GNG12 remain incompletely understood, emerging evidence suggests its involvement in regulating cellular migration^13^, proliferation^14,15^, immunoinflammatory responses^16–18^, and platelet regulation^19–21^. Notably, it has been shown that GNG12 is downregulated across multiple cancer types, suggesting that it may serve as a key regulator in tumorigenesis^22,23^. Moreover, reduced GNG12 expression has been correlated with poor prognosis in several cancers, implying that GNG12 may play a role in tumor progression, though it has not yet been established as a clinical biomarker or therapeutic target^24,25^. Recent studies have further implicated GNG12 in the regulation of cancer cell growth and invasion^26^.

Building on previous studies of GNG12, we conducted a pan-cancer analysis to evaluate GNG12 expression, diagnostic and prognostic significance, functional associations, drug sensitivity, and genomic and epigenetic features. Preliminary functional validation of GNG12 was subsequently performed in TNBC experimental models. Collectively, this study provides a comprehensive characterization of GNG12 expression and its clinicopathological associations across cancers, along with preliminary functional validation of its role in TNBC experimental models.

## Materials and methods

### Differential analysis of GNG12 in pan-cancer

GNG12 expression data were obtained from the harmonized TCGA dataset via the Sparkle database (https://www.grswsci.top/analyze/)^27^. Tumor-specific Z-scores were calculated from the PanCanAtlas file *EBPlusPlusAdjustPANCAN_IlluminaHiSeq_RNASeqV2.geneExp.tsv* using the formula (x − μ)/σ. Outliers with Z-scores falling outside the range of − 3 to 3 were excluded from subsequent analyses. Differential GNG12 expression between tumor and normal tissues was assessed using the Wilcoxon rank-sum test, whereas comparisons between tumor tissues and matched adjacent non-cancerous tissues were performed using the Wilcoxon signed-rank test. At the protein level, GNG12 expression was further analyzed through the UALCAN platform (http://ualcan.path.uab.edu/index.html) based on proteomic data from CPTAC and ICPC^28^. In UALCAN, Z-values represent the number of standard deviations (SD) from the median for each cancer type. CPTAC-derived log2 spectral count ratio values were normalized within individual samples and across the entire dataset.

### The diagnosis and prognostic evaluation of GNG12 in pan-cancer

The prognostic value of GNG12 expression was evaluated using the Kaplan–Meier Plotter online database (https://kmplot.com/analysis/)^29^. Patients were then stratified into high- and low-expression groups based on the platform’s automatically selected optimal cutoff value. Between-group survival differences were compared using Kaplan–Meier analysis and the log-rank test. Hazard ratios (HRs) and 95% confidence intervals (CIs) were derived from univariate Cox proportional hazards models using the platform’s default settings. The diagnostic performance of GNG12 expression was evaluated using TCGA transcriptomic data retrieved from the Sparkle database. Tumor and normal samples were defined according to TCGA annotations. Subsequently, receiver operating characteristic (ROC) analysis was performed in R using the pROC package to assess the discriminatory ability of GNG12 expression, with the area under the ROC curve (AUC) and its 95% CIs also calculated.

### Analysis of the genetic changes of GNG12 in pan-cancer

To characterize GNG12 alterations across cancers, we assessed pan-cancer mutation frequencies using cBioPortal (https://www.cbioportal.org/)^30^. Mutation profiles were retrieved via the “Quick Search” function, and locus-level annotations were subsequently refined using the “Cancer Hotspots” and “Post-translational Modifications (dbPTM)” modules to improve mutation mapping accuracy. Potential phosphorylation sites of the GNG12 protein were predicted using PhosphoNET (http://www.phosphonet.ca/), and phosphorylation patterns in tumors were examined with UALCAN. Predictions from PhosphoNET were then integrated with tumor hotspot information from cBioPortal to identify key residues. These sites were subsequently mapped onto the AlphaFold-predicted structure by activating the “AlphaMissense Pathogenicity” feature, which automatically displays the corresponding amino acid positions on the three-dimensional model.

### Methylation analysis of GNG12 in pan-cancer

Epigenetic regulation of GNG12 was examined by analyzing locus- and cancer type-specific DNA methylation levels using Sparkle, along with promoter methylation analysis via UALCAN. Intergroup methylation differences were assessed using an unpaired two-sample *t*-test. Given the multiple cancer types and loci evaluated in these public-database analyses, the results were considered exploratory unless the corresponding platform performed multiple-testing correction. Associations between GNG12 expression and 44 RNA modification regulators (including genes involved in m1A, m5C, and m6A modification) were evaluated using SangerBox (http://sangerbox.com/home.html)^31^. For correlation analyses involving multiple RNA modification regulators, false discovery rate (FDR) correction was applied when feasible; otherwise, the results were considered exploratory and hypothesis-generating. Uniformly normalized TCGA Pan-Cancer datasets were downloaded from the UCSC Xena Browser (https://xenabrowser.net/), and expression matrices for GNG12 and the 44 RNA modification-related genes were subsequently extracted for analysis. Prior to statistical analysis, all expression values were log2-transformed as log2(x + 0.001).

### Enrichment analysis and single-cell analysis of GNG12 in pan-cancer

Protein–protein interaction (PPI) networks were constructed using STRING. Functional enrichment analyses, including Gene Ontology (GO) and Kyoto Encyclopedia of Genes and Genomes (KEGG) pathway analyses, were then performed with the *clusterProfiler* package in R. Additionally, single-cell functional states were evaluated using CancerSEA (http://biocc.hrbmu.edu.cn/CancerSEA/), a database that annotates 14 cancer-related functional phenotypes across approximately 900 tumor cells from 25 cancer types.

### Drug sensitivity analysis of GNG12 in pan-cancer

Drug sensitivity associated with GNG12 expression was assessed using the Drug module of GSCA^32^. Matched transcriptomic and pharmacological response data were retrieved from the Genomics of Drug Sensitivity in Cancer (GDSC; 265 compounds across 860 cell lines) and the Cancer Therapeutics Response Portal (CTRP; 481 compounds across 1001 cell lines), and the two datasets were then integrated for analysis. Pearson’s correlation analysis was used to evaluate the association between gene expression and IC50 values, and multiple testing correction was then performed using the FDR. For each dataset, the 30 compounds most strongly correlated with GNG12 expression were selected for further analysis. These drug-sensitivity findings were interpreted as exploratory and hypothesis-generating, and require further experimental validation.

### The expression and prognostic analysis of GNG12 in TNBC

Gene expression and clinical information for GNG12 were retrieved from the GEO datasets GSE42568 and GSE9893. These datasets were analyzed independently and without merging with TCGA data, thereby avoiding cross-platform batch effects between microarray and RNA-seq measurements. Differential expression between groups was assessed via the Wilcoxon rank-sum test. Survival analyses were performed using the R survival package, with statistical significance determined by the log-rank test. Associations between GNG12 expression and clinicopathological features were examined in UALCAN using TCGA data; expression differences were visualized as box plots and analyzed by one-way analysis of variance (ANOVA). Spatial expression of GNG12 in TNBC was assessed using Sparkle (BRCA12: GSE210616-GSM6433589; BRCA26: GSE210616-GSM6433603). Micro-regions were categorized as malignant or normal based on deconvolution-derived malignant cell proportions of 1 or 0, respectively. Between-group differences in GNG12 expression were analyzed in R using the Wilcoxon rank-sum test, and mean expression levels were shown as bar plots. Prognostic significance was further validated using GEPIA3 (https://gepia3.bioinfoliu.com/)^33^. Overall survival was estimated via Kaplan–Meier analysis, with significance determined by the log-rank test. HRs and 95% CIs were calculated using a univariate Cox proportional hazards model.

### Clinical specimens

From February 2005 to November 2023, 24 paired samples of TNBC and adjacent non-cancerous breast tissues were collected from the Department of Pathology, the Second People’s Hospital of Lanzhou City. All specimens were independently reviewed and confirmed by two pathologists and subsequently subjected to immunohistochemistry (IHC) analysis. Tissues were fixed in 4% paraformaldehyde (PFA; 26°C, 48 h), embedded in paraffin, and sectioned (5 μm) for subsequent IHC staining. This study was conducted in accordance with the Declaration of Helsinki and approved by the Ethics Committee of the Second People’s Hospital of Lanzhou City. Written informed consent was obtained from all participants before sample collection.

### IHC staining

Paraffin-embedded TNBC sections were deparaffinized, rehydrated, and subjected to antigen retrieval in citrate buffer (pH 6.0). Endogenous peroxidase activity was quenched with 3% hydrogen peroxide, and non-specific binding was blocked with bovine serum. After that, the sections were incubated (overnight, 4 °C) with the primary antibody (Bioss, Beijing, bs-13242R, 1:1000 dilution), followed by incubation (37 °C) with the secondary antibody (Servicebio, GB23303; 1:5000 dilution). Signals were visualized with DAB and counterstained with hematoxylin. Images were acquired using a slide scanner (3DHISTECH, Hungary, CaseViewer2.4). GNG12 expression was quantified as the percentage of DAB-positive area using HALO software (Indica Labs, U.S.A., Halo 101-WL-HALO-1).

### Cells and transfection

The MDA-MB-231 cell line (KCB, Kunming, China; KCB200776Y), a widely used TNBC cell model, was obtained from the Kunming Cell Bank of the Chinese Academy of Sciences. The cells were cultured in Dulbecco’s Modified Eagle Medium/Nutrient Mixture F-12 (DMEM/F-12; Gibco, CA, USA, C11330500BT) supplemented with 10% fetal bovine serum (FBS; Gibco, 10099-141C) and 1% penicillin–streptomycin (Gibco, 15140-122). Cultures were maintained under standard conditions (37 °C, 5% CO_2_, humidified atmosphere).

For lentiviral transduction, MDA-MB-231 cells were infected with either LV17 vectors (carrying GNG12 overexpression constructs or respective negative controls) or LV16 vectors (encoding GNG12-specific shRNA or a non-targeting control) (GenePharma, Shanghai, China). After that, the cells were seeded into 6-well plates and allowed to adhere for 10–12 h before transduction. Transduction was performed in the presence of Polybrene (5 µg/mL) to enhance efficiency. Five experimental groups were established: untreated control (Control), overexpression control (OE-NC), GNG12 overexpression (OE-GNG12), silencing control (sh-NC), and GNG12 knockdown (sh-GNG12). At 72 h post-infection, cells reaching over 90% confluence were transferred to 6-cm culture dishes. Once cultures reached 50% confluence, puromycin (final concentration of 5 µg/mL) was added for selection and maintained in the medium thereafter. The GNG12 shRNA sequence was 5′-ACCCTTTGCTGATAGGAATAC-3′, and the negative control sequence was 5′-TTCTCCGAACGTGTCACGT-3′.

### Western blot analysis

Total cellular proteins were extracted using RIPA lysis buffer (Thermo Scientific™, Shanghai, China, 89900). Protein concentrations were quantified with a BCA protein assay (Thermo Scientific™, 23235). Equal amounts of protein were separated by sodium dodecyl sulfate–polyacrylamide gel electrophoresis (SDS-PAGE) and transferred to PVDF membranes (Biosharp, Anhui, China, BS-PVDF-22). The membranes were blocked (37 °C, 1 h) in TBST containing 5% non-fat milk, followed by incubation (4 °C, overnight) with primary antibodies diluted in the same blocking buffer. After extensive washing with TBST, the membranes were incubated (1 h, room temperature) with horseradish peroxidase (HRP)-conjugated goat anti-mouse IgG (Abmart, Shanghai, China, M21002S, 1:5000) and goat anti-rabbit IgG (Abmart, M21003S, 1:5000) secondary antibodies. Protein bands were detected using a luminol-based ECL substrate (Advansta, CA, USA, K-12045-D50) and imaged with a digital chemiluminescence platform. Band intensities were normalized to GAPDH levels, which served as a loading control. Primary antibodies were: GNG12 (Abcam, UK, AB278545, 1:1000), PI3K (Abmart, T40115S, 1:2000), p-PI3K (Abcam, ab182651, 1:2000), AKT (Abmart, T55561S, 1:3000), p-AKT (Abmart, T40067S, 1:10000), GAPDH (Affinity Biosciences, Jiangsu, China, T0004, 1:1000).

### Cell counting kit-8 (CCK-8) assay

TNBC cells (1 × 10^3^ cells/well) were seeded in 96-well plates (100 μL medium/well) and cultured under standard conditions. At designated time points (24, 48, 72, and 96 h), cells were washed with PBS and incubated with 10 μL CCK-8 solution (Biosharp, BS350B). After incubation for 2 h, the absorbance at 450 nm was measured to determine cell proliferation.

### Wound healing assay

For wound healing assays, TNBC cells were seeded in 6-well plates (1 × 10^5^ cells/well) and grown to a continuous monolayer. A scratch was created using a sterile 100-μL pipette tip, and cells were maintained in serum-free medium. Images of the wound area were captured at 0 and 24 h, and wound closure was quantified using ImageJ software.

### Transwell invasion assays

Cell invasion was assessed using Matrigel (Mogengel, Xiamen, China, 082706)-coated Transwell chambers (Corning, Kennebunk, USA, CLS3422). A suspension containing 5 × 10^3^ cells in serum-free medium (200 μL) was placed in the upper chamber, and medium containing 10% FBS (600 μL) was added to the lower chamber as a chemoattractant. After incubation (37 °C, 24 h), the inserts were washed with PBS, fixed with 4% PFA, and stained (10 min) with 0.1% crystal violet (Beyotime, Shanghai, China, CO121). Non-invading cells on the upper surface were removed, and cells that invaded through the membrane were counted under a microscope.

### In vivo xenograft model

Female BALB/c-Nude mice (4 weeks, 16–18 g) obtained from the Lanzhou Veterinary Research Institute (Chinese Academy of Agricultural Sciences) were used to establish subcutaneous xenografts. TNBC cells with stable GNG12 knockdown or overexpression constructs (5 × 10⁶ cells mixed 1:1 with Matrigel) were injected into the dorsal flank of each mouse. Mice were randomly allocated into five groups (n = 6/group): Control, sh-NC, sh-GNG12, OE-NC, and OE-GNG12. Tumor volume was measured weekly for 7 weeks, after which tumors were collected. Four mice per group with successfully established measurable tumors, complete serial tumor-volume records, and available tumor tissues were included in the final analysis. Mice were excluded from the final analysis if any of the following criteria were met: (1) no measurable tumor formed; (2) serial tumor-volume measurements were incomplete; and (3) tumor tissues were unavailable due to modeling failure. At the end of the experiment, all animals were humanely euthanized by cervical dislocation under deep 0.3% pentobarbital sodium anesthesia, in accordance with the approved IACUC protocol. The study was conducted in accordance with ARRIVE guidelines and approved by the Ethics Committee of the Lanzhou Veterinary Research Institute, Chinese Academy of Agricultural Sciences.

### Statistical analyses

The data accessed through Sparkle, UALCAN, GEPIA3, and SangerBox were all derived from the TCGA database. These tools were selected based on their use of harmonized TCGA data with documented normalization pipelines, each assigned a non-overlapping analytical role corresponding to a distinct data type (mRNA expression, protein expression, methylation, or RNA modification). To minimize potential batch effects, analyses were performed within individual datasets or platforms, and results from different resources were compared qualitatively rather than by direct integration of raw expression matrices. GEO validation cohorts were analyzed independently using their respective normalized expression data. Therefore, potential inter-platform variability was minimized through dataset-specific analyses and independent cross-cohort validation. For public database analyses involving multiple comparisons, FDR correction was applied when available or when specified by the analytical platform. Analyses without multiple-testing correction were considered exploratory and hypothesis-generating rather than confirmatory. Experimental replicates refer to independent biological replicates unless otherwise specified. In vitro experiments were independently repeated at least three times. Results are presented as mean ± SD. Statistical analyses were performed using the Wilcoxon signed-rank test for paired comparisons, the Wilcoxon rank-sum test for unpaired comparisons, and one-way ANOVA for comparisons among multiple groups. Repeated tumor-volume measurements were analyzed using two-way repeated-measures ANOVA, with appropriate post hoc comparisons performed where applicable. For IHC quantification of paired TNBC and adjacent non-cancerous tissues, differences were assessed using the Wilcoxon signed-rank test. Survival outcomes were evaluated via the Kaplan–Meier method and the log-rank test. A two-sided *p-*value < 0.05 was considered statistically significant.

## Results

### GNG12 is downregulated in pan-cancer

**Fig. 1:**
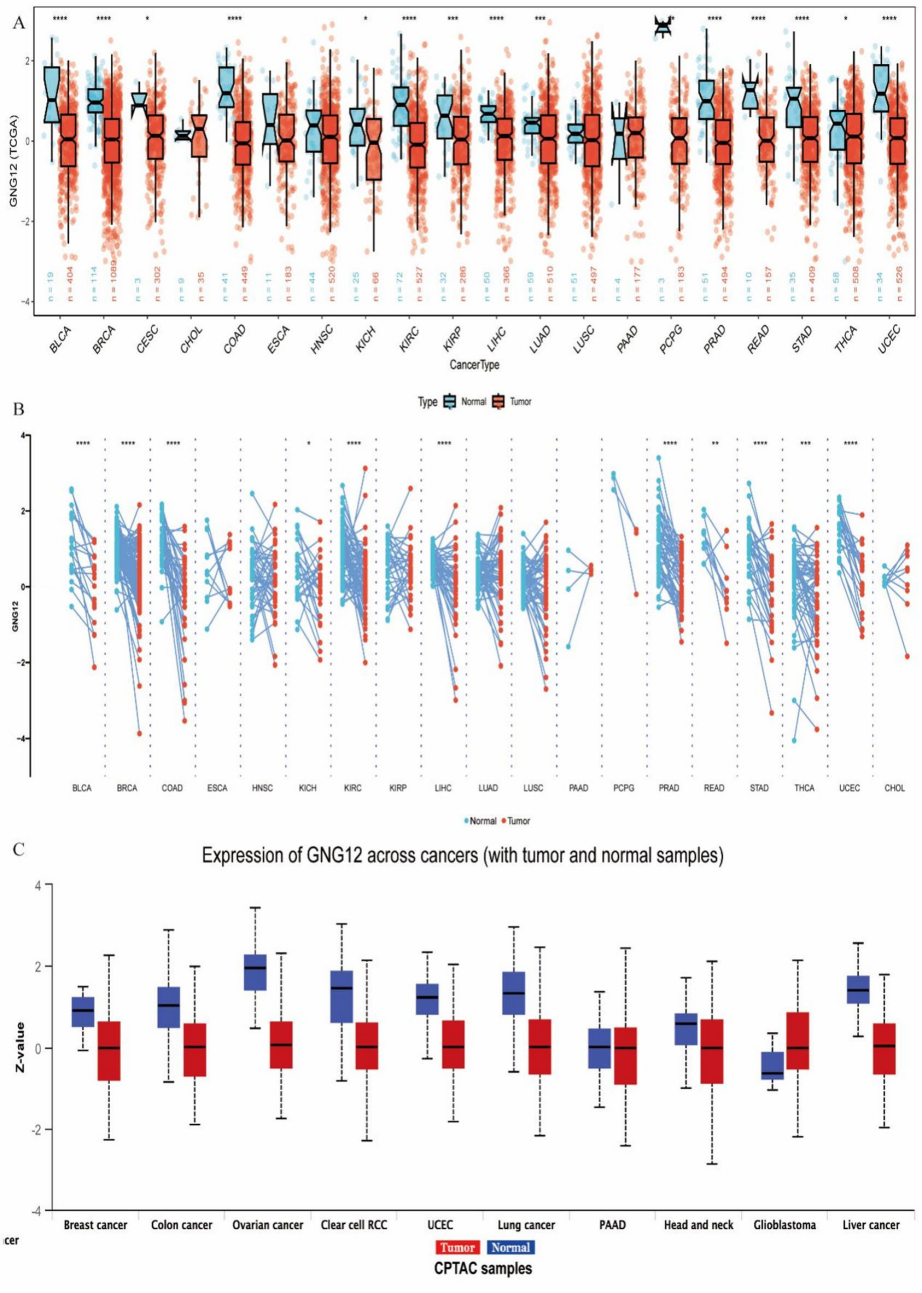
Expression patterns of GNG12 at mRNA and protein levels in pan-cancer. (**A**) GNG12 mRNA expression in pan-cancer. (**B**) Pair tumor-normal comparison of GNG12 mRNA expression in pan-cancer. (**C**) GNG12 protein level in pan-cancer.

To characterize the expression pattern of GNG12 across human malignancies, we analyzed TCGA transcriptomic datasets. GNG12 mRNA expression was reduced in 15 tumor types relative to adjacent normal tissues (Fig. 1A). This reduction was further confirmed by paired analyses in 11 cancer types (Fig. 1B). Similarly, proteomic profiling of clinical specimens also revealed decreased GNG12 protein abundance in eight tumor types (Fig. 1C). Taken together, these results identified GNG12 as a widely downregulated gene across diverse cancers at both the transcriptional and protein levels.

### GNG12 expression is associated with clinical features in pan-cancer

**Fig. 2:**
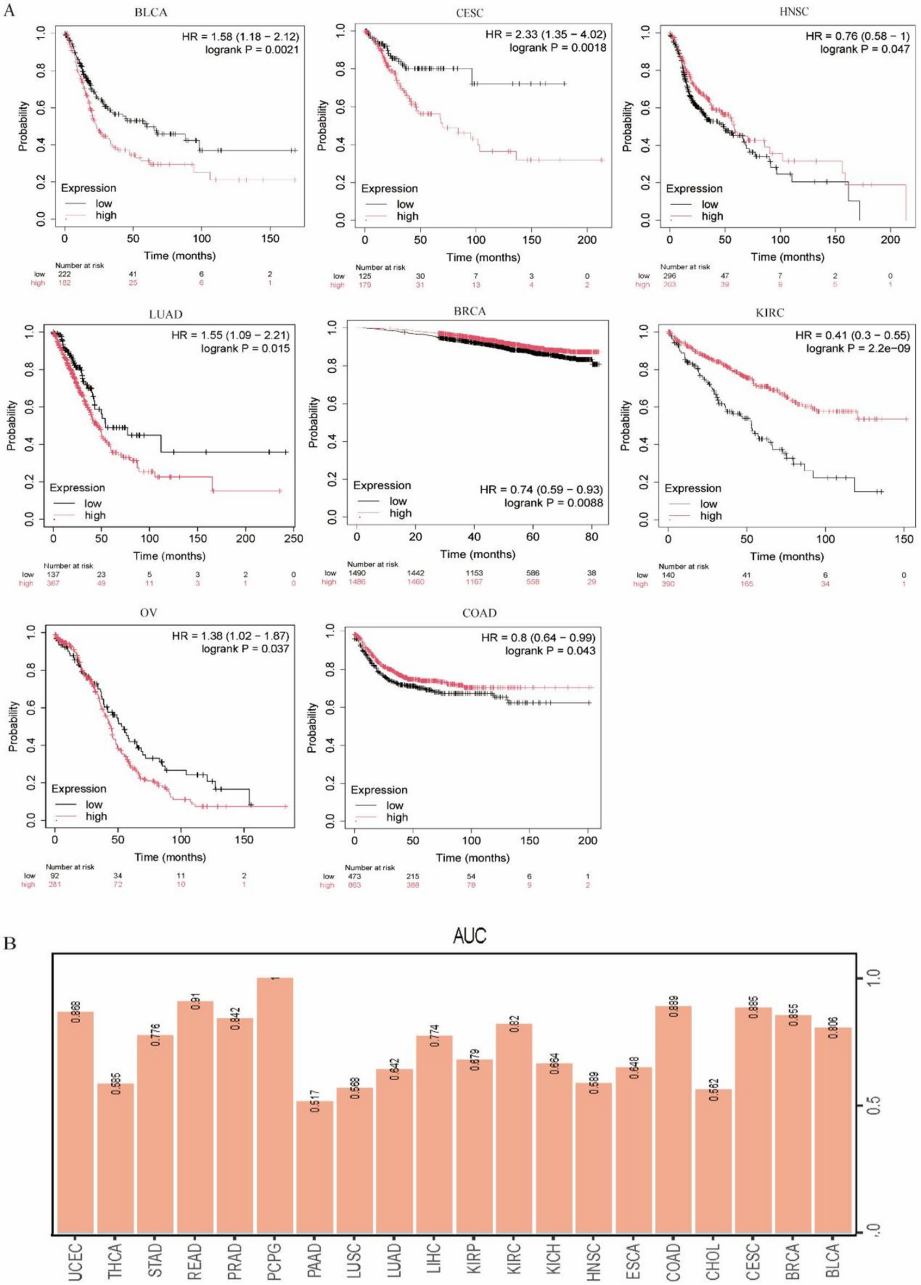
Diagnostic and prognostic significance of GNG12 in pan-cancer. (**A**) Kaplan–Meier survival curves stratified by GNG12 expression. (**B**) ROC analysis of GNG12 for distinguishing tumor from normal tissues.

To investigate the clinical relevance of GNG12 in human cancers, we assessed its association with patient survival using the Kaplan–Meier Plotter tool. GNG12 expression was significantly associated with overall survival in eight cancer types: BLCA (Bladder Cancer), CESC (Cervical Cancer), HNSC (Head and Neck Cancer), KIRC (Kidney Clear Cell Carcinoma), OV (Ovarian Cancer), COAD (Colon Cancer), LUAD (Lung Adenocarcinoma), and BRCA (Fig. 2A). Notably, lower GNG12 expression was consistently associated with poorer outcomes in three of these cancer types: KIRC, COAD, and BRCA. ROC analysis was further performed to determine whether GNG12 expression differed between tumor and normal tissues. The results showed strong discriminatory ability in PCGC (Pheochromocytoma & Paraganglioma), PAAD (Pancreatic Cancer), UCEC (Endometrioid Cancer), and BRCA (Fig. 2B). Together, these findings indicated that GNG12 expression was associated with both survival outcomes and the ability to distinguish tumor tissues from normal tissues across multiple cancer types.

### GNG12 has genetic variants in pan-cancer

Somatic mutations can reshape cellular signaling and drive tumor progression, affecting proliferation and metastatic potential. In our pan-cancer analysis, GNG12 exhibited genetic alterations in 21 of 32 cancer types examined (Figure S1A and Supplementary Material 3). Among the recurrent variants, T68 substitutions were observed in stomach adenocarcinoma, breast invasive lobular carcinoma, and colon adenocarcinoma, while S33 mutations were enriched in uterine endometrioid carcinoma (Supplementary Material 1). Given the role of post-translational modifications in protein regulation, we next examined the PTM profile of GNG12. Phosphorylation emerged as the most frequently predicted modification, followed by acetylation, ubiquitination, methylation, palmitoylation, and S-nitrosylation (Figure S1B and Supplementary Material 3). Several residues were identified as PTM-sensitive sites, including position 2 (associated with both phosphorylation and acetylation) and position 68 (linked to methylation and palmitoylation). We further predicted 11 phosphorylation sites in GNG12 (S2, S3, T5, S7, T17, S26, S33, S41, Y42, S49, and S59). Of these, seven sites (S2, S3, T5, S26, S41, Y42, and S49) have been experimentally validated in mammalian systems, supporting their potential as phosphorylation sites (Figure S1C and Supplementary Material 3). S49-phosphorylated GNG12 was reduced in multiple tumor types (Figure S2A and Supplementary Material 3). By contrast, S7, T17, S33, and S59 have not yet been experimentally confirmed, highlighting the need for further validation. cBioPortal analysis revealed that S2 and S33 are residues present in both mutational and phosphorylation events, suggesting that these positions may serve as regulatory hotspots. Residues predicted to be highly pathogenic are mapped in red, including S2 and S33 (Figure S2B and Supplementary Material 3). In silico mutational analysis further showed that the G2A substitution exhibited moderate pathogenicity (score = 0.330) and moderate structural confidence (pLDDT = 61.86). In contrast, the S33G > A substitution displayed high pathogenicity (score = 0.910) and high structural confidence (pLDDT = 98.49), suggesting a greater likelihood of functional relevance (Figure S2C and Supplementary Material 3).

### GNG12 displays differential methylation patterns in pan-cancer

To explore the relationship between GNG12 and epigenetic mechanisms, we analyzed promoter DNA methylation and RNA methylation-related signals. GNG12 exhibited differential methylation patterns across tumor types and genomic loci (Figure S3A and Supplementary Material 3). Promoter methylation was lower in CHOL and THCA compared with normal tissues, whereas higher levels were observed in BRCA, UCEC, READ, LIHC, COAD, HNSC, and ESCA (Figure S3B and Supplementary Material 3). Furthermore, GNG12 expression was positively correlated with RNA methylation regulators in most cancers (Figure S3C and Supplementary Material 3), reinforcing a broad association between GNG12 expression and epigenetic regulation.

### Enrichment analysis and single-cell profiling reveal GNG12-related mutations across pan-cancer

**Fig. 3:**
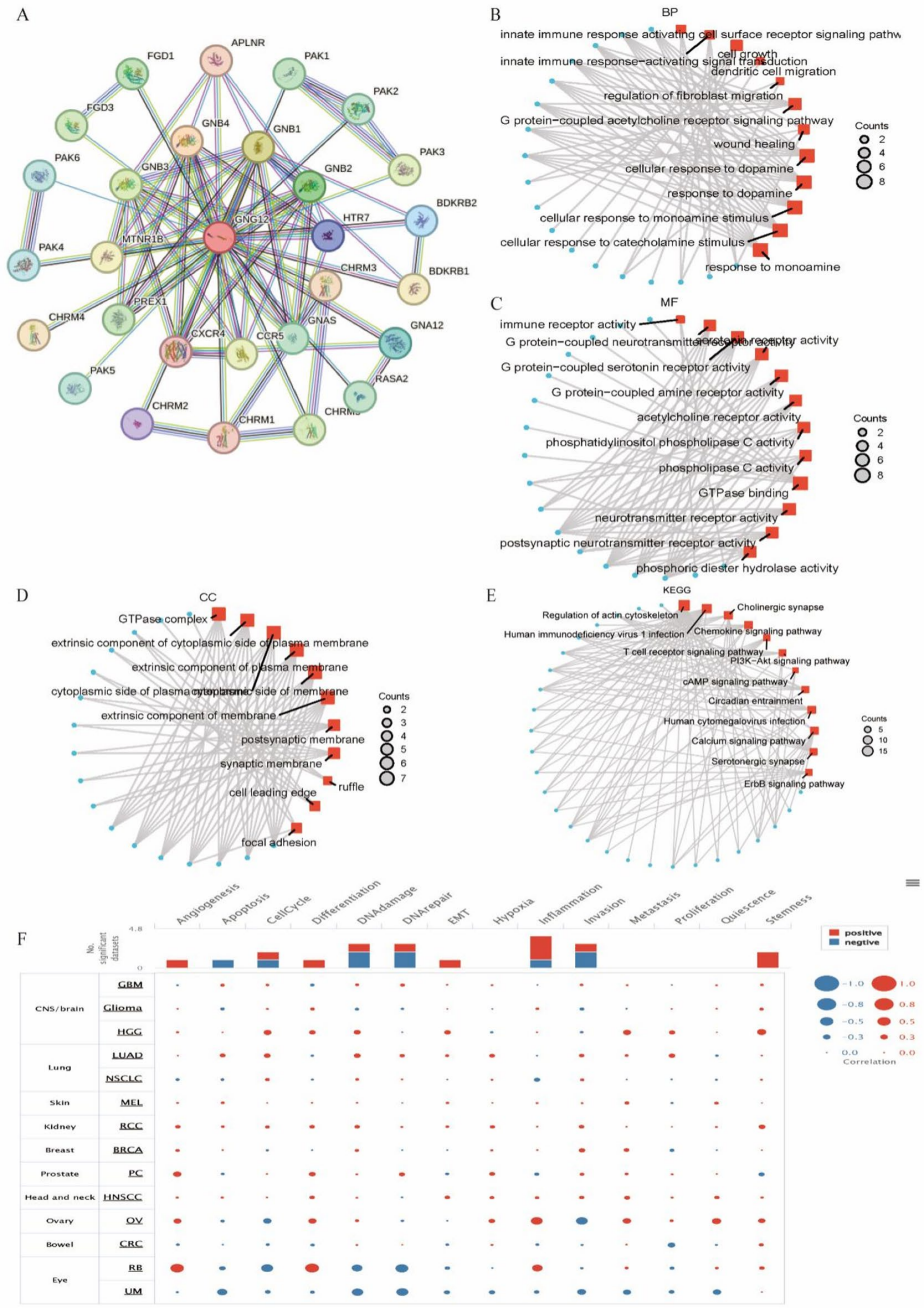
Enrichment analysis and single-cell analysis of GNG12. (**A**) PPI network of GNG12 and its interacting genes. (**B**–**D**) GO enrichment analysis of GNG12-related genes. (**E**) KEGG pathway enrichment analysis of GNG12-related genes. (**F**) Single-cell functional analysis of GNG12. The Kanehisa Laboratory granted permission for the result of using KEGG and the following KEGG images.

A STRING-based PPI network was constructed to investigate the molecular context of GNG12 in tumor progression, and 28 putative interacting proteins were identified (Fig. 3A). GO analysis revealed significant enrichment in processes related to innate immune activation, cell growth control, dendritic cell migration, wound healing, fibroblast migration, and G protein-coupled acetylcholine receptor signaling (Fig. 3B). GNG12 was mainly localized to heterotrimeric G-protein and GTPase complexes and the cytoplasmic face of the plasma membrane (Fig. 3C), and its molecular functions were linked to immune and neurotransmitter receptor activity (Fig. 3D). KEGG analysis further highlighted pathways including actin cytoskeleton regulation, Ras and PI3K-Akt signaling, and HIV-1 infection (Fig. 3E). Additionally, CancerSEA analysis revealed that GNG12 was associated with epithelial-mesenchymal transition (EMT), metastasis, invasion, DNA damage response, proliferation, apoptosis, inflammation, and differentiation at the single-cell level (Fig. 3F).

### GNG12 expression is associated with drug sensitivity in pan-cancer

Analysis of drug response data from the CTRP and GDSC databases revealed significant associations between GNG12 expression and sensitivity to multiple therapeutic compounds (Figure S4A, B and Supplementary Material 3). These findings suggested that GNG12 may be related to differential drug responsiveness; however, these results should be interpreted as exploratory and hypothesis-generating, and require further experimental validation.

### GNG12 is downregulated in TNBC

**Fig. 4:**
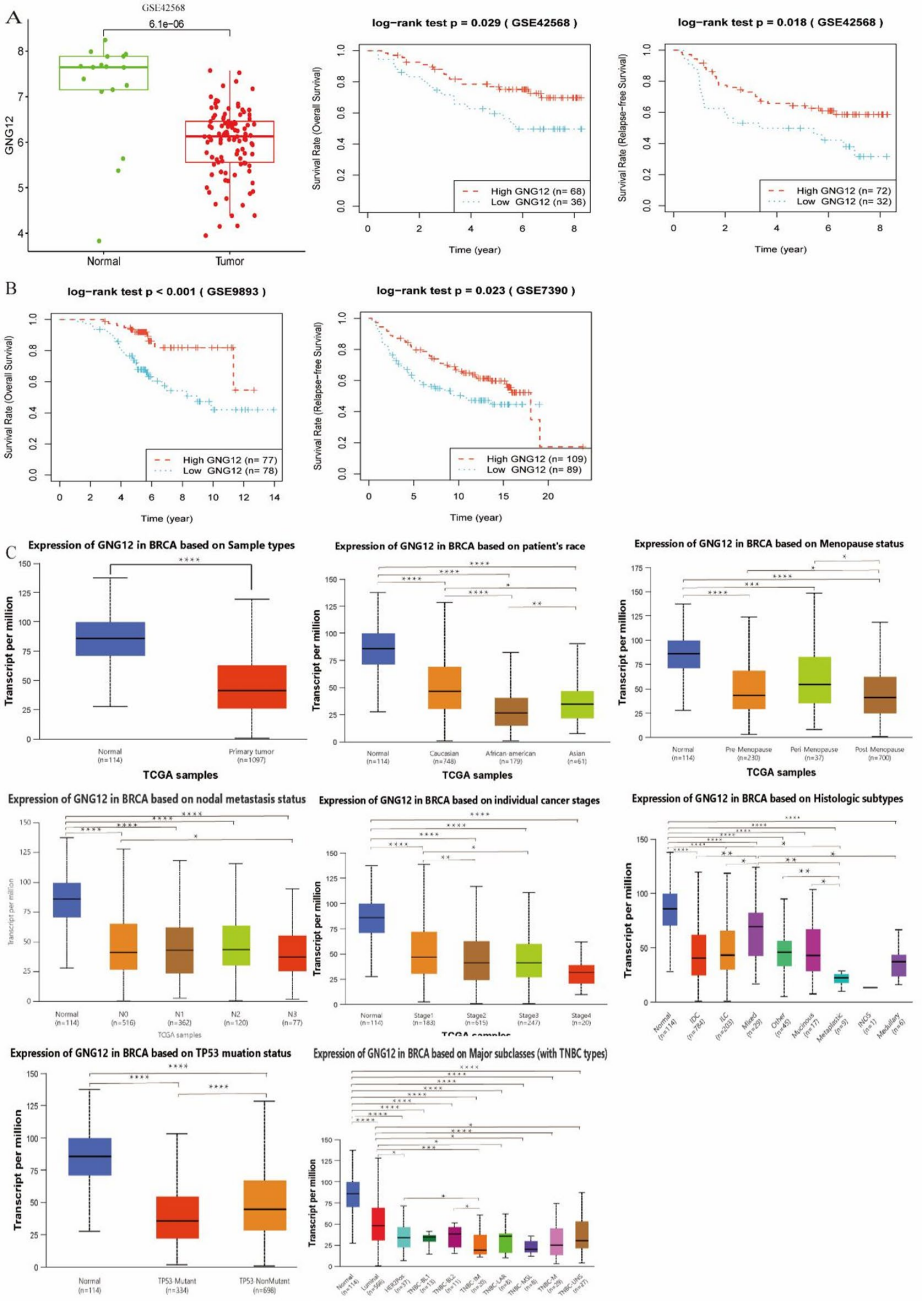
GNG12 expression and prognosis in TNBC. (**A**) GNG12 expression and overall survival/relapse-free survival analysis in the GSE42568 dataset. (**B**) Overall survival and relapse-free survival analysis of GNG12 in the GSE9893 dataset. (**C**) Association between GNG12 expression and clinicopathological features of BRCA in BRCA (patient race, menopause status, nodal metastasis, tumor stage, histologic subtype, TP53 mutation status, and molecular subclassification).

**Fig. 5:**
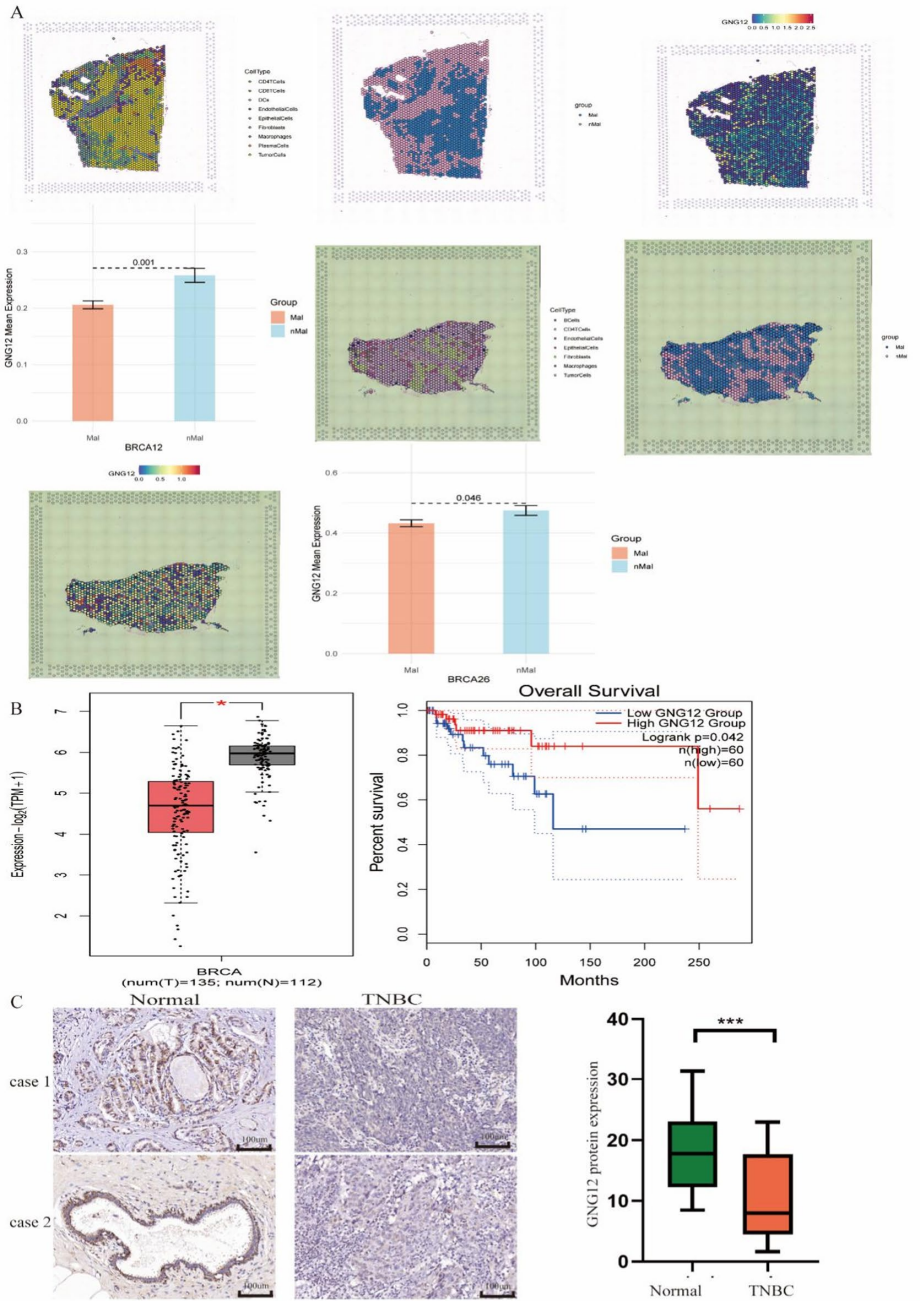
GNG12 expression and prognosis in TNBC. (**A**) The distribution and expression of GNG12 in both malignant (Mal) and non-malignant (nMal) cells. (**B**) Expression and prognosis of GNG12 in TNBC. (**C**) GNG12 expression in clinical TNBC tumor tissues and normal tissues.

Given that low GNG12 expression is associated with poor prognosis in BRCA from our pan-cancer analyses, we next investigated the role of GNG12 in BRCA. Analysis of the GSE42568 and GSE9893 cohorts confirmed the differential expression pattern of GNG12 and its prognostic relevance in BRCA (Fig. 4A, B). Clinicopathological correlation analysis further demonstrated significant associations between reduced GNG12 expression and race, menopausal status, nodal metastasis, tumor stage, histological subtype, TP53 mutation status, and molecular subclassification (Fig. 4C). Spatial transcriptomic analysis in TNBC further revealed markedly lower GNG12 expression in malignant regions compared with non-malignant tissues (Fig. 5A). Consistently, GEPIA3 analysis showed that low GNG12 expression was associated with poor prognosis in TNBC (Fig. 5B). To further validate these findings at the protein level, IHC was performed on 24 paired TNBC tumor and adjacent non-cancerous tissue samples from the Lanzhou Second People’s Hospital (Supplementary Material 2). The results showed that GNG12 IHC scores were significantly lower in tumor tissues than in matched adjacent non-cancerous tissues (Wilcoxon signed-rank test; Fig. 5C), further confirming GNG12 downregulation in TNBC.

### GNG12 overexpression suppresses the proliferative and metastatic capacities of TNBC

**Fig. 6:**
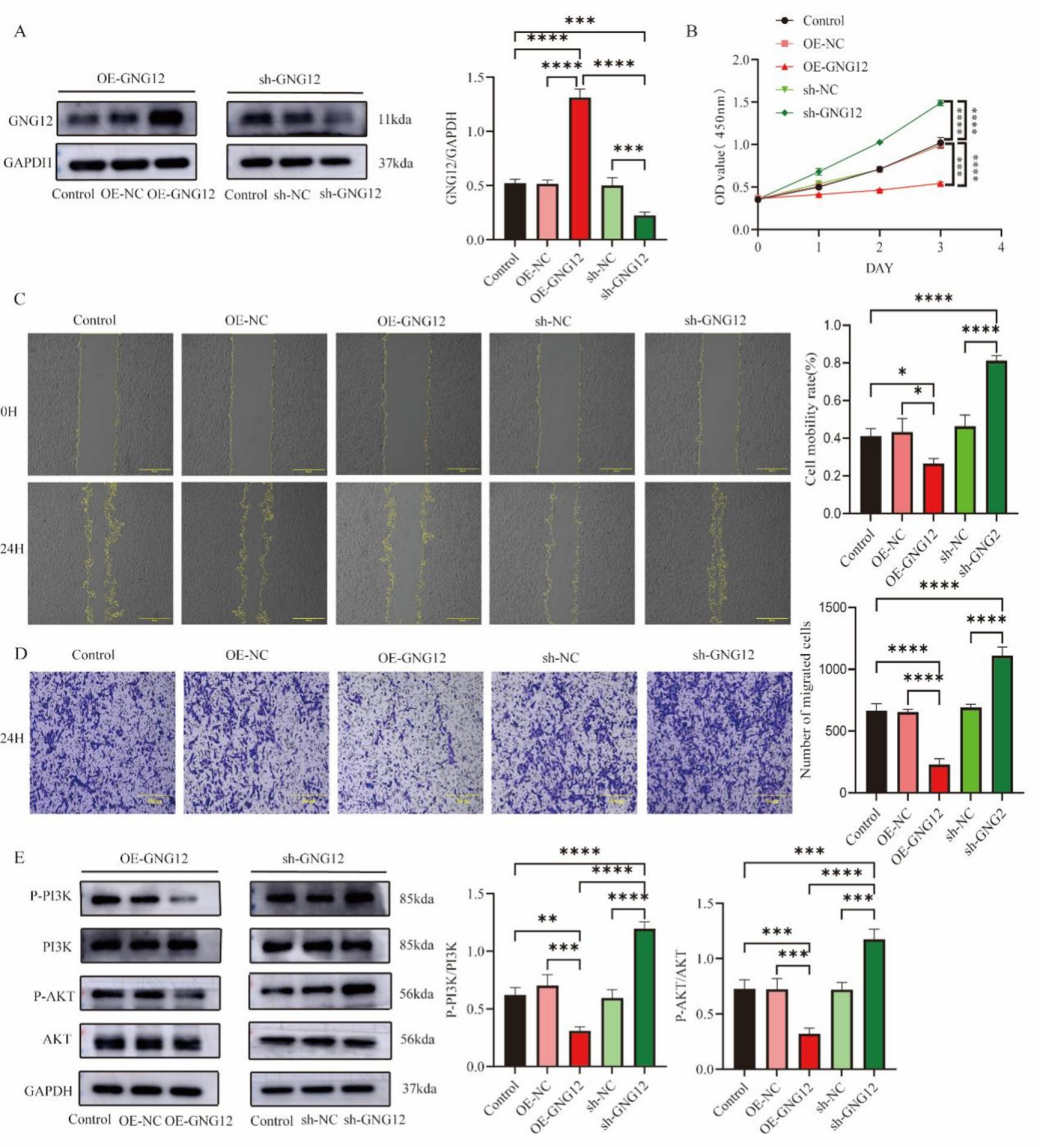
GNG12 overexpression suppresses malignant cellular phenotypes in TNBC cells. (**A**) GNG12 expression after OE-GNG12 and sh-GNG12 transfection. (**B**) Effects of GNG12 overexpression and knockdown on TNBC cell growth. (**C**) Effects of GNG12 overexpressionand knockdown on cell migratory behavior. (**D**) Effects of GNG12 overexpression and knockdown oncell invasive capacity. (**E**) Expression and phosphorylation levels of the PI3K/AKT pathway.

Our results showed that decreased GNG12 expression was associated with poorer clinical outcomes in TNBC. To investigate the functional role of GNG12, we both silenced and overexpressed GNG12 in MDA-MB-231 cells (Fig. 6A). As revealed by CCK-8 assays, GNG12 overexpression notably inhibited TNBC cell proliferation, whereas GNG12 knockdown promoted TNBC cell growth (Fig. 6B). According to the wound healing assay results, GNG12 overexpression markedly reduced the migratory ability of TNBC cells, while GNG12 silencing enhanced cell migration (Fig. 6C). Similarly, Transwell invasion assays showed that GNG12 overexpression suppressed the invasive capacity of TNBC cells, whereas GNG12 knockdown exerted the opposite effects (Fig. 6D). These findings indicated that GNG12 may function as a suppressor of TNBC cell proliferation, migration, and invasion. Since KEGG analysis suggested enrichment of GNG12 in the PI3K/AKT pathway, pathway activation was further analyzed using western blotting. It was found that the phosphorylation levels of PI3K and AKT were significantly reduced in cells overexpressing GNG12; in contrast, GNG12 knockdown increased the phosphorylation levels of PI3K and AKT (Fig. 6E).

### GNG12 overexpression is associated with reduced xenograft growth in vivo

**Fig. 7:**
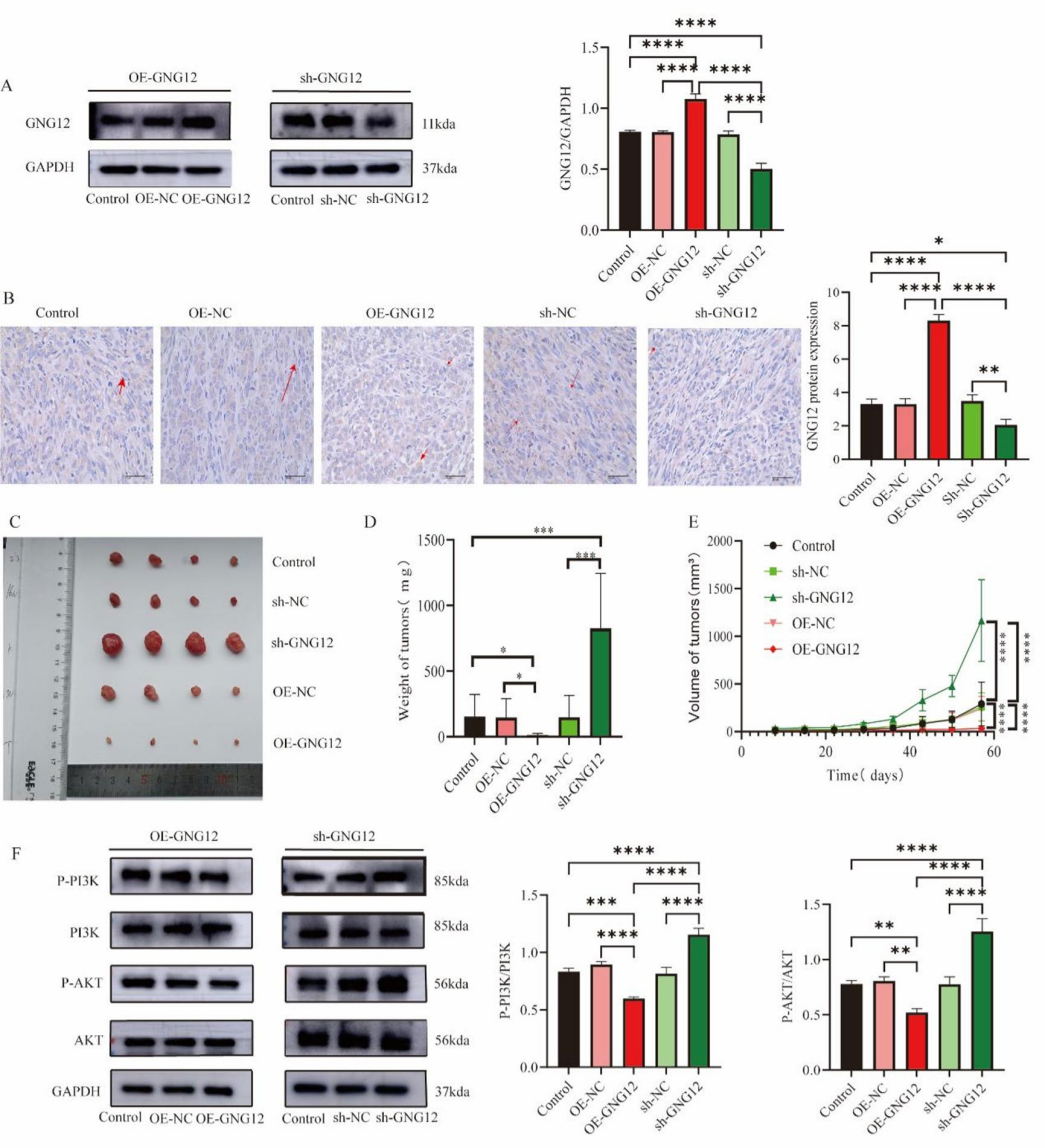
GNG12 overexpression is associated with reduced xenograft growth in vivo. (**A**) GNG12 expression in xenografts with GNG12 overexpression and knockdown. (**B**) Representative IHC staining of GNG12 in xenografts. (**C**) Representative images of xenograft tumors. (**D**) Tumor growth curves. (**E**) Tumor weights. (**F**) Expression and phosphorylation levels of the PI3K/AKT pathway in xenografts.

Based on the in vitro findings that GNG12 expression suppressed malignant phenotypes of TNBC cells, its role was further examined in vivo using xenograft models with GNG12 overexpression or knockdown (Fig. 7A, B). Specifically, six mice were initially assigned to each group, and four mice per group with measurable tumors and complete records were included in the final analysis. Serial measurements of tumor volume showed that GNG12 overexpression reduced xenograft growth, whereas GNG12 silencing promoted tumor progression (Fig. 7C–E). Consistent with the in vitro observations, GNG12 overexpression was associated with reduced PI3K/AKT pathway activity in xenograft tumors (Fig. 7F). Conversely, GNG12 depletion correlated with increased PI3K/AKT pathway activity.

## Discussion

Our pan-cancer and experimental data collectively suggest that GNG12 may exert tumor-suppressive effects across human cancers^24,25^. Although previous studies have reported reduced GNG12 expression in specific malignancies, including BRCA^34^, LUAD^35^, UCEC^36^, and COAD^37^, its pan-cancer expression pattern has not been fully characterized. By integrating transcriptomic and proteomic evidence, our analysis demonstrated that GNG12 was predominantly downregulated in most cancer types, suggesting a potential association between GNG12 downregulation and tumor progression. The observed associations between low GNG12 expression and poor survival, particularly in KIRC, COAD, and BRCA, further suggested that GNG12 may be a marker of unfavorable prognosis. However, these findings are mainly based on public database analyses and should be interpreted as exploratory rather than evidence of established clinical utility. The mechanistic basis underlying GNG12 dysregulation and its functional consequences in different tumor contexts remains to be further explored.

Accumulating studies have shown that genetic and epigenetic alterations are central to tumor initiation and progression^38,39^. In this context, our bioinformatic analyses identified several potentially relevant mutation sites in GNG12 that may affect protein structure and function, thereby modulating cancer-related processes. Notably, phosphorylation appeared to be an important regulatory mechanism. This finding is consistent with prior evidence that GNG12 is linked to kinase-associated signaling pathways that regulate cell proliferation, survival, and metastatic behavior^11^. More specifically, two mutation hotspots, S2 and S33, overlapped with putatively pathogenic predicted phosphorylation sites, suggesting that these residues may be functionally important in tumor biology. In addition, we observed cancer-type-specific differences in GNG12 promoter methylation, suggesting that DNA methylation may contribute to its dysregulated expression in malignancy^40^. We further examined associations between GNG12 expression and major RNA methylation regulators, given the established role of aberrant RNA methylation in cancer. These analyses may offer preliminary insights into the epigenetic regulation of GNG12^41,42^. Finally, significant correlations were observed between GNG12 expression and sensitivity to several chemotherapeutic agents. These findings, together with prior evidence that paclitaxel can modulate GNG12 expression, should be considered exploratory and hypothesis-generating, and further experimental and clinical studies are needed to determine therapeutic response^43^.

Our analyses revealed that GNG12 expression was significantly associated with multiple clinicopathological characteristics in BRCA, and its expression decreased progressively with advancing disease severity, with the lowest levels detected in TNBC samples^44^. These observations suggest that GNG12 may be involved in BRCA progression, particularly in TNBC. Single-cell RNA sequencing and functional enrichment analyses further revealed that GNG12 was associated with invasion, metastasis, EMT, proliferation, and apoptosis. Consistently, similar patterns have also been reported in other malignancies, including glioblastoma (GBM) and PAAD, where GNG12 appears to be associated with proliferative, migratory, and invasive phenotypes^45,46^. To further explore the functional significance of GNG12 in TNBC, we performed gain-of-function experiments and found that GNG12 overexpression suppressed TNBC cell proliferation, migration and invasion, supporting that GNG12 may play a potential tumor-suppressive role in this context. KEGG pathway analysis further identified the PI3K–AKT signaling pathway as a potential candidate mediator of GNG12-related functional changes. Given the established role of PI3K-AKT signaling in TNBC progression, this result may have biological relevance. Previous studies have also implicated GNG12 in GBM through the same pathway^47–50^. Consistently, proteins associated with the PI3K-AKT signaling were downregulated in the GNG12-overexpression model. However, the current findings are insufficient to establish a direct regulatory relationship between GNG12 and the PI3K-AKT signaling. It remains possible that GNG12 acts indirectly through upstream factors or broader G protein-mediated signaling networks.

In summary, our data suggested that decreased GNG12 expression was associated with unfavorable clinical outcomes in multiple malignancies, an association particularly evident in TNBC. Functional experiments further indicated that GNG12 overexpression suppressed TNBC cell proliferation, migration, and invasion, supporting a potential tumor-suppressive role for GNG12. In addition, our pathway analyses suggested the involvement of the PI3K/AKT signaling, although further studies are needed to determine whether this pathway is directly regulated by GNG12. These findings suggested that GNG12 was associated with TNBC progression and malignant cellular phenotypes. Nevertheless, its clinical relevance and mechanistic relationship with the PI3K/AKT signaling require further validation in larger clinical cohorts and additional experimental models.

Nevertheless, Several limitations should be considered when interpreting these results. First, the use of multiple public databases and analytical platforms represents a potential concern. Although Sparkle, GEPIA3, and SangerBox use standardized data with documented normalization pipelines, residual differences in preprocessing and analytical strategies may still introduce bias. To mitigate this issue, transcriptomic, methylation, and proteomic analyses were conducted within their respective platforms rather than by directly integrating raw datasets. In addition, GEO cohorts were analyzed independently and used only for external validation. Despite these efforts, residual heterogeneity among public datasets cannot be entirely excluded and should be considered when interpreting the results. Second, our results demonstrate an association between GNG12 and PI3K/AKT signaling, but do not establish a causal relationship. Further studies, such as mechanistic investigations of candidate upstream regulators, protein interaction analyses, and pathway-rescue experiments, will be needed to determine whether GNG12 directly modulates the PI3K/AKT signaling or acts through indirect regulatory mechanisms. Third, the experimental validation is limited to a small number of TNBC models. Additional cell lines, patient-derived models, and larger clinical cohorts are required to further evaluate the biological and clinical significance of GNG12.

## Supplementary Information

Below is the link to the electronic supplementary material.

Supplementary Material 1

Supplementary Material 2

Supplementary Material 3

Supplementary Material 4

Supplementary Material 5

Supplementary Material 6

## Data Availability

This study analyzed publicly available datasets, which can be accessed at the following locations: https://www.grswsci.top/analyze/, http://ualcan.path.uab.edu/index.html, https://kmplot.com/analysis/, https://www.cbioportal.org/, http://www.phosphonet.ca/, https://alphafold.com/, http://sangerbox.com/home.html, https://cn.stringdb.org/, http://biocc.hrbmu.edu.cn/CancerSEA/, http://sangerbox.com/home.html, https://gepia3.bioinfoliu.com/, and https://www.ncbi.nlm.nih.gov/geo/.

## Abbreviations

ACC: Adrenocortical cancer
BLCA: Bladder cancer
BRCA: Breast cancer
CESC: Cervical cancer
CHOL: Bile duct cancer
COAD: Colon cancer
COADREAD: Colon and rectal cancer
DLBC: Large B-cell lymphoma
ESCA: Esophageal cancer
GBM: Glioblastoma
GBMLGG: Lower grade glioma and glioblastoma
HNSC: Head and neck cancer
KICH: Kidney chromophobe
KIRC: Kidney clear cell carcinoma
KIRP: Kidney papillary cell carcinoma
LAML: Acute myeloid leukemia
LGG: Lower grade glioma
LIHC: Liver cancer
LUAD: Lung adenocarcinoma
LUNG: Lung cancer
LUSC: Lung squamous cell carcinoma
MESO: Mesothelioma
OV: Ovarian cancer
PAAD: Pancreatic cancer
PANCAN: Pan-cancer
PCPG: Pheochromocytoma & paraganglioma
PRAD: Prostate cancer
READ: Rectal cancer
SARC: Sarcoma
SKCM: Melanoma
STAD: Stomach cancer
TGCT: Testicular cancer
THCA: Thyroid cancer
THYM: Thymoma (THYM)
UCEC: Endometrioid cancer
UCS: Uterine carcinosarcoma
UVM: Ocular melanomas
