# Supplementary material for: GNG12 overexpression inhibits triple-negative breast cancer cell proliferation, migration, and invasion
Source: Sci Rep. 2026 Aug 11;16:25896. doi: 10.1038/s41598-026-66202-z (PMC13486845; doi:10.1038/s41598-026-66202-z)
Supplement: Supplementary file 1 [file 41598_2026_66202_MOESM3_ESM.docx]

**Supplementary Information**

Figure S1 Genetic of GNG12 in pan-cancer. A: Genetic alterations in GNG12 expression across cancers. B: Annotated sites of epigenetic modifications. C: Phosphorylation sites of GNG12.

Figure S2 Genetic characterization of GNG12 in pan-cancer. A: Phosphorylation analysis of GNG12. B: Pathogenicity heatmap of GNG12. C: Distribution of S33 and S2 on the three-dimensional structural diagram

Figure S3 Methylation analysis of GNG12 A: Differences in GNG12 methylation levels across distinct genetic loci. B: Methylation levels of GNG12 across pan-cancer types. C: Relationship between GNG12 and RNA methylation-related genes.

Figure S4. Drug sensitivity analysis of GNG12. A: GNG12 expression positively correlates with various targeted or chemotherapeutic agents in the GDSC database. B: GNG12 expression correlates with multiple targeted or chemotherapeutic compounds in the CTRP database
