# Supplementary material for: GNG12 overexpression inhibits triple-negative breast cancer cell proliferation, migration, and invasion
Source: Sci Rep. 2026 Aug 11;16:25896. doi: 10.1038/s41598-026-66202-z (PMC13486845; doi:10.1038/s41598-026-66202-z)
Supplement: Supplementary file 2 [file 41598_2026_66202_MOESM6_ESM.docx]

Supplementary materials

[Supplementary Material 1](https://pmc.ncbi.nlm.nih.gov/articles/instance/12639060/bin/41598_2025_26188_MOESM1_ESM.docx): Detailed list of GNG12 mutations across various cancers

[Supplementary Material 2](https://pmc.ncbi.nlm.nih.gov/articles/instance/12639060/bin/41598_2025_26188_MOESM1_ESM.docx): Clinical information of 24 TNBC cases

[Supplementary Material 3](https://pmc.ncbi.nlm.nih.gov/articles/instance/12639060/bin/41598_2025_26188_MOESM1_ESM.docx): Supplementary figures

Supplementary Material 4: Full-length, uncropped images of all bands on the Western blot membranes. The membranes were cut according to molecular weight markers prior to antibody incubation; the intact edges of each membrane are clearly visible in the images.

Supplementary Material 5: KEGG License
